# Supplementary material for: Neuroanatomy in a middle Cambrian mollisoniid and the ancestral nervous system organization of chelicerates
Source: Nat Commun. 2022 Jan 20;13:410. doi: 10.1038/s41467-022-28054-9 (PMC8776822; doi:10.1038/s41467-022-28054-9)
Supplement: Supplementary file 1 — Supplementary Information [file 41467_2022_28054_MOESM1_ESM.pdf]

Supplementary Information

Neuroanatomy in a middle Cambrian mollisoniid and the ancestral nervous system organization of chelicerates

Javier Ortega-Hernández[1,*], Rudy Lerosey-Aubril[1], Sarah R. Losso[1], and James C. Weaver[2]

[1]Museum of Comparative Zoology and Department of Organismic and Evolutionary Biology, Harvard University, Cambridge, MA 02138, USA.

[2]Wyss Institute for Biologically Inspired Engineering, 60 Oxford Street, Harvard University, Cambridge, MA 02138, USA.

*Corresponding author: jortegahernandez@fas.harvard.edu

Supplementary Figures

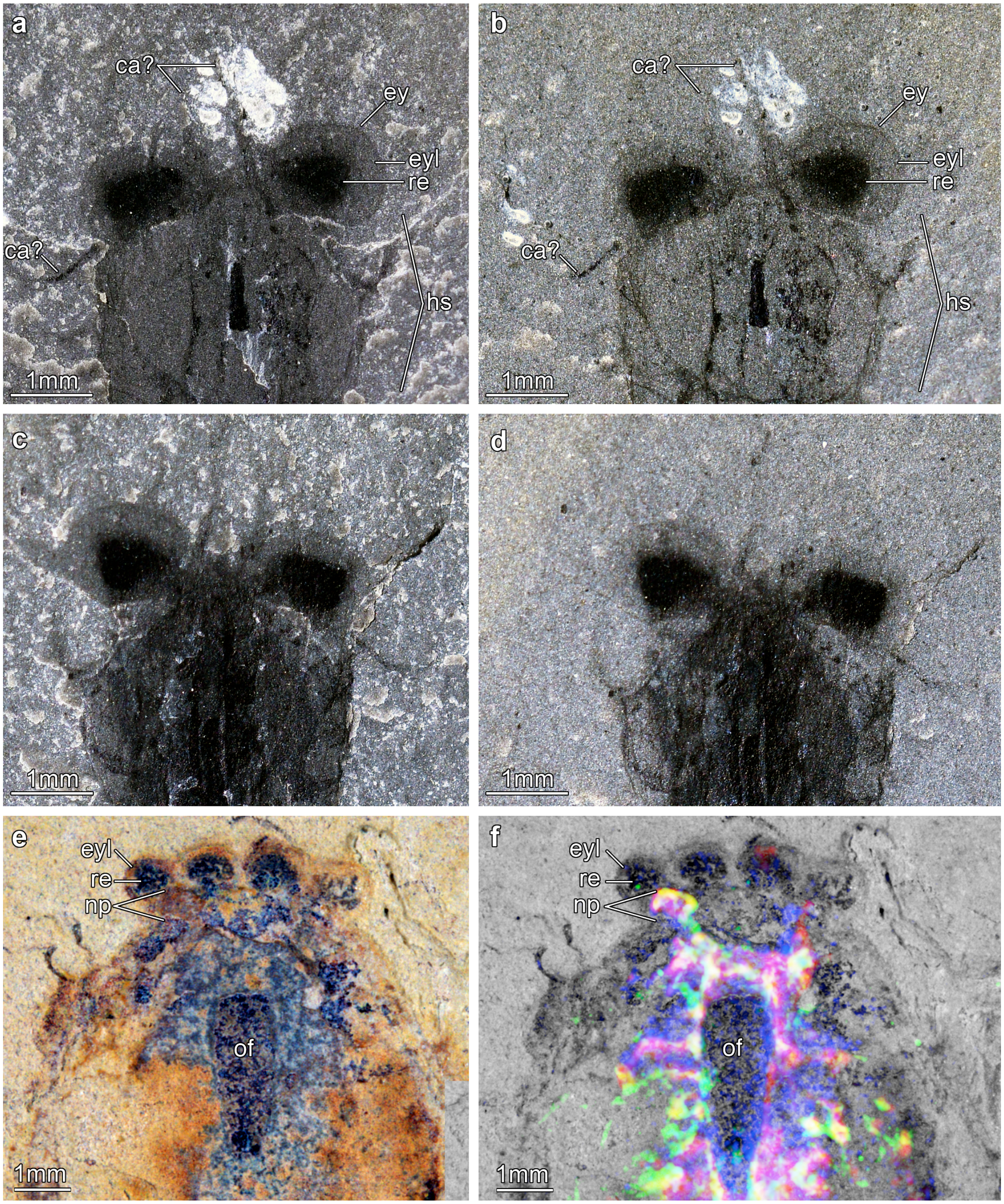

Supplementary Figure 1. Comparison of cephalic morphology between the Burgess Shale *Mollisonia symmetrica* and the Chengjiang *Alalcomenaeus* sp. a. MCZ 1811a, photographed under polarized illumination, dry. **b.** MCZ 1811a, photographed under polarized illumination, wet. **c.** MCZ 1811b, photographed under polarized illumination, dry. **d.** MCZ 1811a, photographed under polarized illumination, wet. e. *Alalcomenaeus* sp. (Yunnan Key Laboratory for Palaeobiology no. 11075) photographed under reflected light. f. YKLP 11075 showing superimposition of micro-CT scan, Fe and Cu elemental maps (reproduced from ref. [1]). Abbreviations: *ca?,* putative cephalic appendage; *ey*, lateral eyes; *eyl*, eye lens; *g*, gut tract; *h*s, head shield; *np*, optic neuropil; *of*, oesophageal foreman; *re*, retina; *Tn*, trunk tergite.

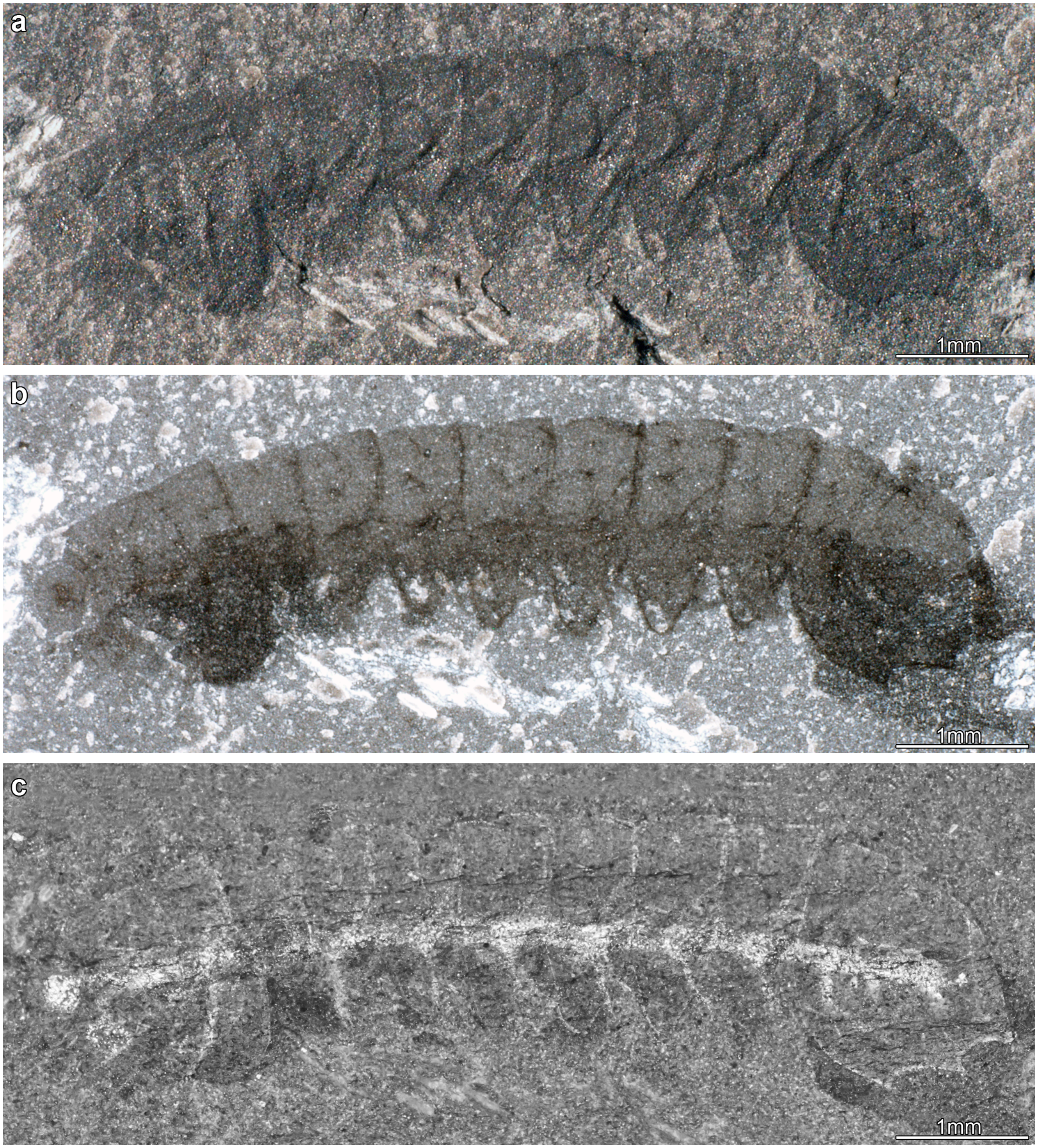

Supplementary Figure 2. Central nervous system preservation in *Mollisonia symmetrica* from the Cambrian (Wuliuan) Burgess Shale, Walcott Quarry, without annotations. a. USNM 305093, photographed dry with low angle reflected light to enhance topographic information; arrowheads indicate position of spines in pygidium. **b.** USNM 305093, photographed dry with cross-polarized illumination to enhance specimen outline. **c.** USNM 305093, photographed underwater with cross-polarized illumination to highlight morphology of central nervous system.

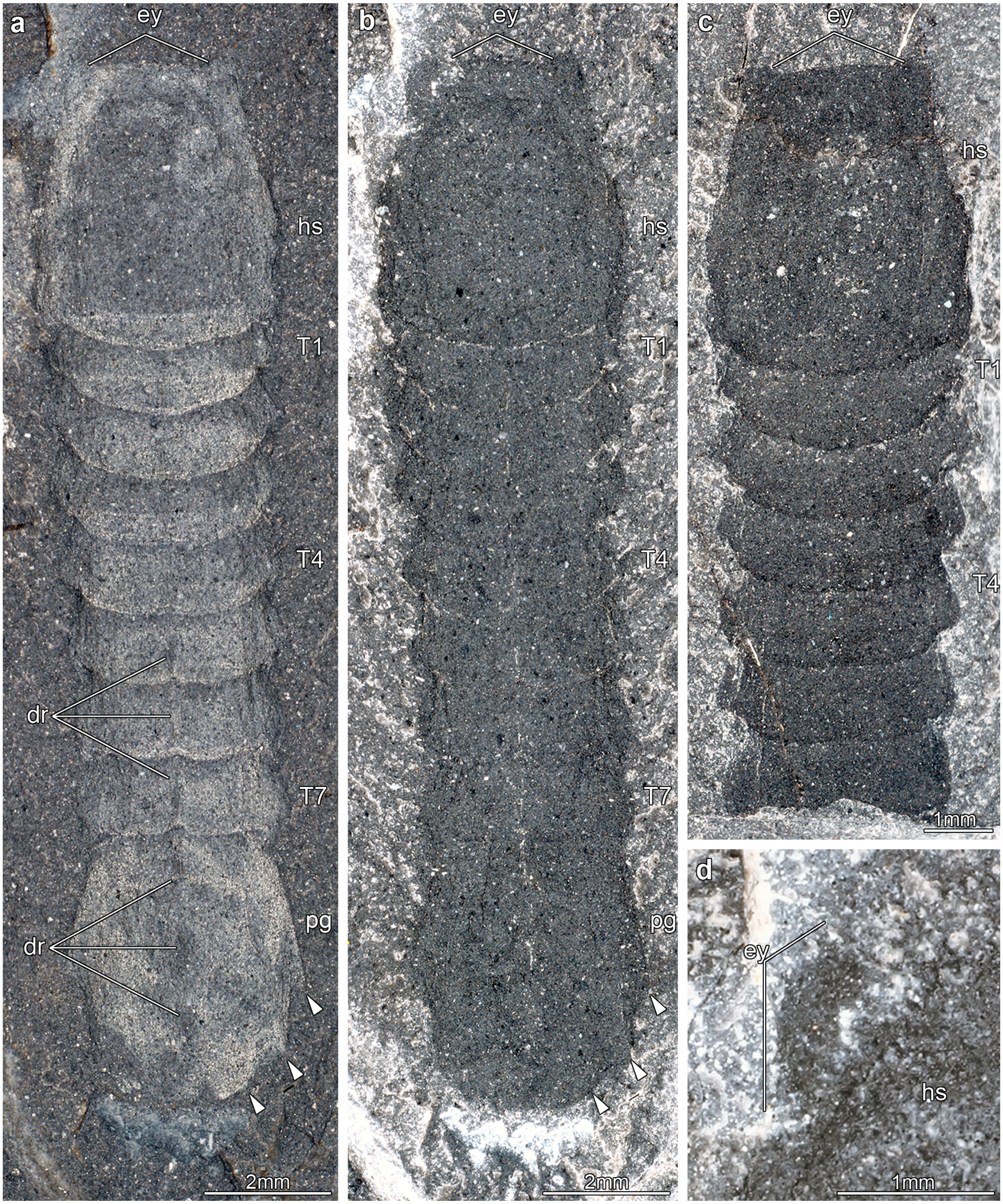

Supplementary Figure 3. ***Mollisonia symmetrica*** **juvenile from the Cambrian (Wuliuan) Burgess Shale. a.** USNM 57661a, photographed underwater with cross-polarized illumination; arrowheads indicate position of spines in pygidium. Note that specimen USNM 57661 was initially classified as the holotype of Mollisonia gracilis Walcott (see main text for details). **b.** USNM 57661a, photographed dry with cross-polarized illumination. **c.** USNM 57661b, photographed dry with cross-polarized illumination. **d.** USNM 57661a, detail of preserved lateral eye. Abbreviations: *dr,* dorsal ridge; *ey,* lateral eyes; *hs,* head shield; *pg*, pygidium; *Tn,* trunk tergite.

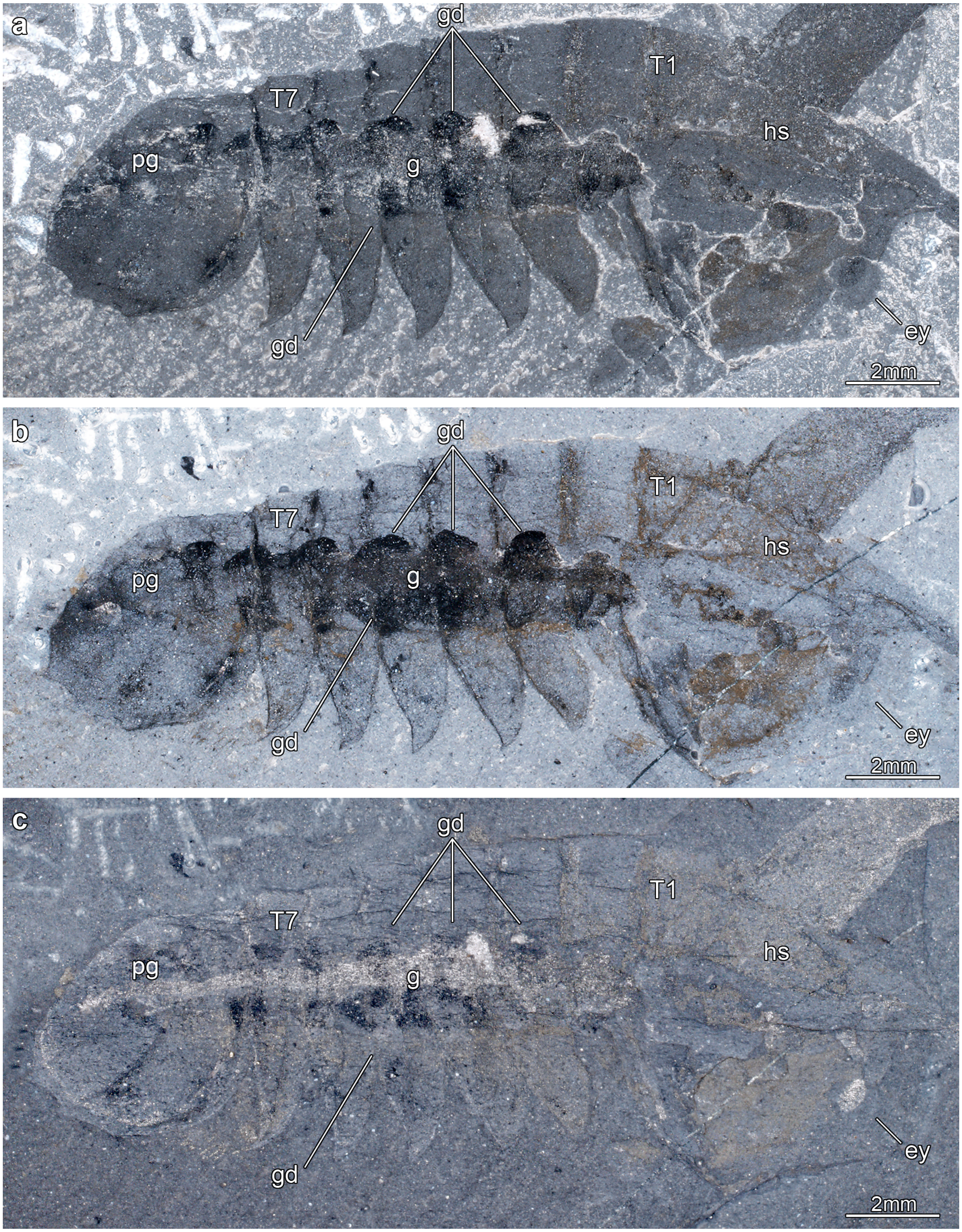

Supplementary Figure 4. Gut tract and diverticula preservation in *Mollisonia* from the Cambrian (Wuliuan) Burgess Shale. a. USNM 57663, photographed dry with cross-polarized illumination**. b.** USNM 57663, photographed wet with direct illumination; note presence of serially repeated and robust phosphatized gut diverticula. **c.** USNM 57663, photographed wet with cross-polarized illumination; note gut tract highlighted as a wide reflective film associated with paired gut diverticula. Abbreviations*: ey,* lateral eyes*; g,* gut tract; *gd,* gut diverticula; *hs,* head shield*; pg,* pygidium; *Tn,* trunk tergite.

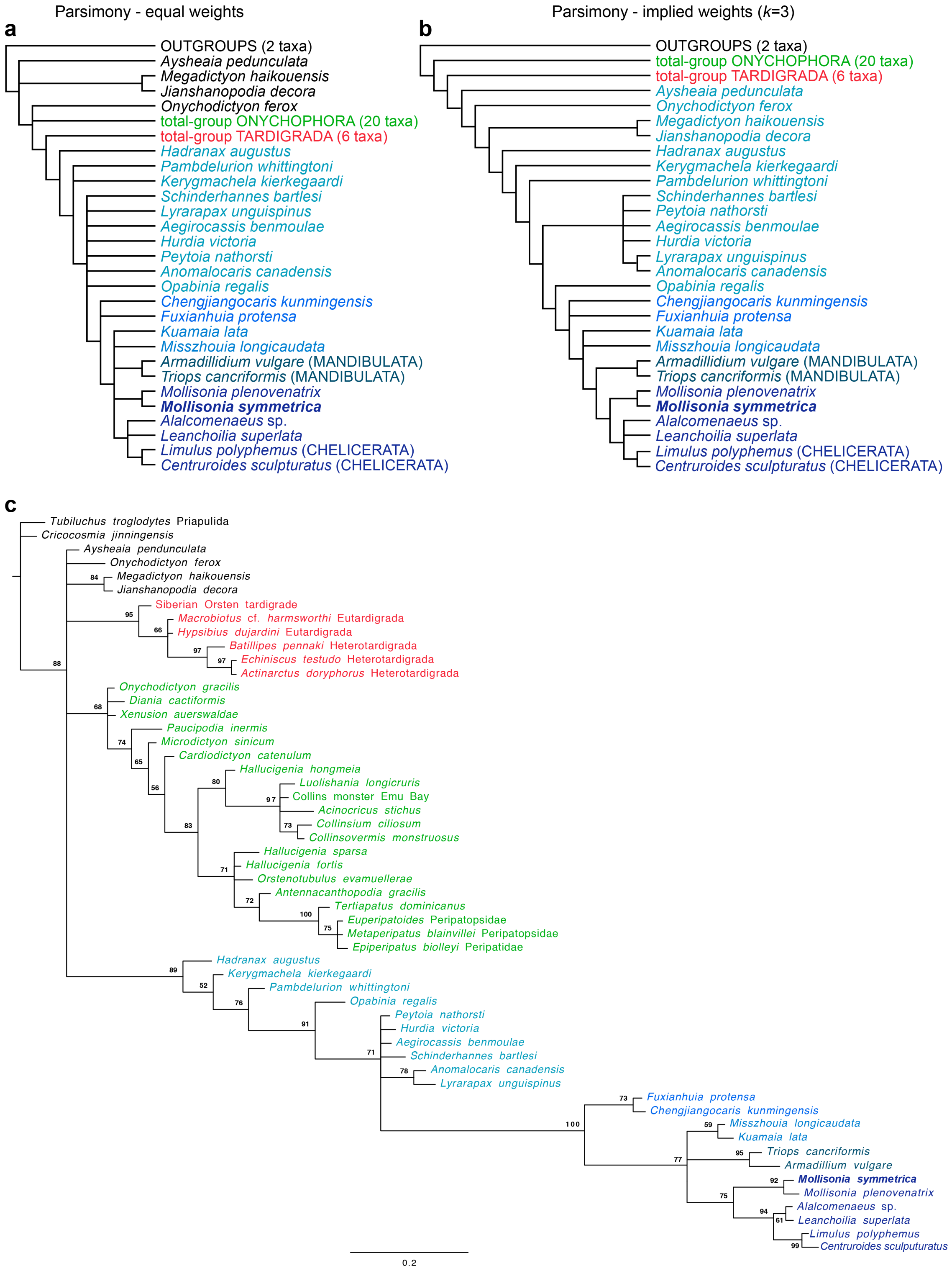

Supplementary Figure 5. Summary of results of phylogenetic analysis of 54 taxa and 106 morphological characters. a. Maximum parsimony, strict consensus of 515 most parsimonious trees (MPTs) under equal weights (205 steps; CI = 0.64, RI = 0.88). **b.** Maximum parsimony, strict consensus of 166 MPTs under implied weights (k = 3; CI = 0.63, RI = 0.88). **c.** Consensus tree resulting from Bayesian analysis in MrBayes. Mk model, four chains, 1,000,000 generations, 1/1000 sampling resulting in 1000 samples, 25% burn-in resulting in 750 samples retained. Numbering denotes posterior probability values.

Supplementary Notes

The dataset used for the phylogenetic analysis has been updated from that used by Yang et al. 2016 [2] to explore evolutionary relationships among Cambrian panarthropods, with emphasis on the implementation of fossilized neuroanatomical data. The new dataset includes our findings on *Mollisonia symmetrica,* as well as the appendicular information on the closely related *Mollisonia plenovenatrix* [3] that has been used to support chelicerate affinities for mollisoniids. We have also incorporated relevant neuroanatomical characters from the dataset used by Tanaka et al. [1], which focuses on the megacheiran *Alalcomenaeus* sp. For a detailed discussion of character scoring under a developmentally-informed model that reflects the fundamental change in head organization observed from lower to upper stem-group Euarthropoda (*sensu* ref. 4, 5), see Smith and Ortega-Hernández [6], Yang et al. 2015 [7], and Yang et al. 2016 [2].

General organization

1. Paired appendages
(0) absent
(1) present

Character 1 in Yang et al. [7].

Head region

2. Anterior region covered by sclerites
(0) absent
(1) present

Character 2 in Yang et al. [7].

3. Head shield formed by fused cephalic segments
(0) absent
(1) present
(–) inapplicable: head sclerites (Character 2) absent

Character 3 in Yang et al. [7].

4. Isolated dorsal sclerite associated with eye-stalks
(0) absent
(1) present
(–) inapplicable: head sclerites (Character 2)

Character 4 in Yang et al. [7].

5. Shape of dorsal isolated sclerite
(0) semicircular/rounded
(1) well-developed and elongate
(–) inapplicable: isolated dorsal sclerite (Character 4) absent

Character 5 in Yang et al. [7].

6. Extent of coverage of dorsal isolated sclerite on head
(0) broad attachment to cephalic region
(1) narrow attachment with anterior edge of cephalic region
(–) inapplicable: isolated dorsal sclerite (Character 4) absent

Character 6 in Yang et al. [7].

7. Isolated lateral sclerites, forming tri-partite carapace
(0) absent
(1) present
(–) inapplicable: isolated dorsal sclerite (Character 4) absent; cephalic shield (Character 3) present

Character 7 in Yang et al. [7].

8. Anterolateral notches in cephalon accommodating eyes [NEW]
(0) absent
(1) present
(–) inapplicable: cephalic shield (Character 3) absent

Anterolateral cephalic notches accommodating a set of well-developed lateral eyes are expressed in all mollisoniids [8].

9. Cephalic/anterior appendages
Nature of post-ocular (post-protocerebral) body appendages
(0) lobopodous
(1) arthropodized (sclerotized; arthrodial membranes present)
(–) inapplicable: paired appendages (Character 1) absent

Character 8 in Yang et al. [7].

10. Sclerotization of pre-ocular (protocerebral) limb pair
(0) not sclerotized
(1) sclerotized
(–) inapplicable: paired appendages (Character 1) absent

Character 9 in Yang et al. [7].

11. Pre-ocular (protocerebral) limb pair with arthrodial membranes
(0) absent
(1) present
(–) inapplicable: protocerebral limbs (Character 10) not sclerotized

Character 10 in Yang et al. [7].

12. Nature of post-ocular lobopodous inner branch
(0) cylindrical/subconical appendage
(1) laterally expanded swimming flap
(–) inapplicable: post-ocular limbs, if present, are arthropodized (Character 9)

Character 11 in Yang et al. [7].

13. Nature of first post-ocular (deutocerebral) appendage
(0) lobopodous ambulatory limb
(1) lobopodous sensorial limb
(2) loboodous limb with sclerotized jaw
(3) arthropodized antenniform with distinct podomeres
(4) arthropodized short great-appendage/chelicerae
(–) inapplicable: paired appendages (Character 1) absent

Character 12 in Yang et al. [2].

14. Inner blade of deutocerebral jaw with diastema
(0) absent
(1) present
(–) inapplicable: deutocerebral jaw (Character 13) absent

Character 13 in Yang et al. [7].

15. Deutocerebral limb pair structurally differentiated from rest of trunk appendages
(0) undifferentiated, or reduced in size only
(1) structurally differentiated
(–) inapplicable: paired appendages (Character 1) absent

Character 14 in Yang et al. [7].

16. Nature of second post-ocular (tritocerebral) appendage
(0) undifferentiated lobopodous limb
(1) specialized papillae
(2) arthropodized ambulatory limb with distinct podomeres
(3) arthropodized specialized post-antennal appendage
(4) arthropodized biramous second antennae
(5) arthropodized chelate pedipalp
(-) inapplicable: paired appendages (Character 1) absent

Character 15 in Yang et al. [2].

17. Position of pre-ocular (protocerebral) appendage pair
(0) lateral
(1) ventral
(2) terminal
(–) inapplicable: paired appendages (Character 1) absent

Character 16 in Yang et al. [7].

18. Pre-ocular (protocerebral) appendage pair fused
(0) not fused
(1) fused
(–) inapplicable: paired appendages (Character 1) absent

Character 17 in Yang et al. [7].

19. Nature of pre-ocular (protocerebral) appendage fusion
(0) basal only, with separate distal elements
(1) fused into a labrum
(–) inapplicable: protocerebral appendages (Character 18) not fused

Character 18 in Yang et al. [7].

20. Labrum greatly reduced, inconspicuous in adult form [NEW]
(0) absent
(1) present
(–) inapplicable: protocerebral appendages (Character 19) not fused into labrum

A reduced labrum has been recently confirmed for the megacheiran *Leanchoilia illecebrosa* [9], which is morphologically and topologically similar to the condition observed in extant euchelicerates.

21. Spines/spinules on pre-ocular (protocerebral) appendage
(0) absent
(1) present (radiodontans, gilled lobopodians, certain lobopodians)
(–) inapplicable: paired appendages (Character 1) absent; protocerebral appendages fused into labrum (Character 20)

Character 19 in Yang et al. [7].

22. Number of spine/spinule series on pre-ocular (protocerebral) frontal appendage
(0) one series (e.g. Aysheaia, Kerygmachela, Opabinia)
(1) two series (e.g. Anomalocaris canadensis, Onychodictyon ferox, Schinderhannes bartelsi)
(–) inapplicable: protocerebral appendages fused into labrum (Character 20); spines/spinules on the protocerebral appendages (Character 21) absent

Character 20 in Yang et al. [7].

23. Coplanar spine/spinule series in pre-ocular (protocerebral) frontal appendages
(0) no
(1) yes
(–) inapplicable: protocerebral appendages fused into labrum (Character 20); spine/spinules, if present, in single series (Character 22)

Character 21 in Yang et al. [7].

24. Multifurcate distal termination of protocerebral appendage
(0) absent
(1) present
(–) inapplicable: protocerebral appendages fused into labrum (Character 20); spines/spinules on the protocerebral appendages (Character 24) absent

Character 22 in Yang et al. [7].

Oral structures

25. Mouth opening orientation
(0) anterior
(1) ventral
(2) posterior

Character 23 in Yang et al. [7].

26. One or more pairs of appendages located anteriorly relative to the mouth opening
(0) absent
(1) present
(-) innaplicable; paired appendages (Character 1) absent

Character 24 in Yang et al. [7].

27. Radially symmetrical circumoral structures
(0) absent
(1) present

Character 25 in Yang et al. [7].

28. Nature of radial circumoral structures
(0) scalids
(1) oral papillae or lamellae
(2) radial plates organized as a mouth apparatus
(–) inapplicable: radial circumoral structures (Character 27) absent

Character 26 in Yang et al. [7].

29. Structure of mouth apparatus
(0) variable number of undifferentiated plates (e.g. *Pambdelurion*)
(1) plates with differentiation of three or four enlarged plates (i.e. *Radiodonta*)
(–) inapplicable: circumoral structures, if present, are not radial plates (Character 28)

Character 27 in Yang et al. [7].

30. Inner rows of teeth within mouthpart
(0) absent
(1) present
(–) inapplicable: circumoral structures, if present, are not radial plates (Character 28)

Character 28 in Yang et al. [7].

Ocular structures

31. Eyes
(0) absent
(1) present

Character 29 in Yang et al. [7].

32. Eye attachment
(0) eye sessile
(1) eye stalked
(–) inapplicable: eyes (Character 31) absent

Character 30 in Yang et al. [7].

33. Type of eyes
(0) single lens eye or pigment spots
(1) multiple visual units (including compound eyes)
(–) inapplicable: eyes (Character 31) absent

Character 31 in Yang et al. [7].

34. Lateral eye doublets [NEW]
(0) absent
(1) present
(–) inapplicable: eyes (Character 31) absent

Eye doublets are expressed in leanchoiliid megacheirans [9], as well as extant *Limulus polyphemus* [1].

35. Nature of eye doublets [NEW]
(0) homonomous (all eyes are compound)
(1) heteronomous (compound eyes and median single lens eye)
(-) inapplicable: eye doublets (Character 34) absent

Leanchoiliid megacheirans have homonomous eye doublets consisting of only compound eyes (or eyes with multiple visual units) [9], whereas extant euchelicerates have heteronomous doublets, consisting of lateral compound eyes and median single lens eyes [1].

Trunk region

36. Epidermal segmentation
(0) absent
(1) present

Character 32 in Yang et al. [7].

37. Dorsal integument sclerotized and connected by arthrodial membranes
(0) absent
(1) present

Character 33 in Yang et al. [7].

38. Sternites connected by arthrodial membranes
(0) absent
(1) present
(–) inapplicable: dorsal sclerotized integument (Character 37) absent

Character 34 in Yang et al. [7].

39. Dorsal exoskeleton parallel sided [NEW]
(0) absent
(1) present
(–) inapplicable: dorsal sclerotized integument (Character 37) absent

A parallel-sided dorsal exoskeleton is a synapomorphy of mollisoniids [8].

40. Neck-like constriction on lobopodous trunk
(0) absent
(1) present
(–) inapplicable: sclerotized dorsal integument with arthrodial membranes (Character 37) present

Character 35 in Yang et al. [7].

41. Annulations
(0) absent
(1) present
(–) inapplicable: sclerotized dorsal integument with arthrodial membranes (Character 37) present

Character 36 in Yang et al. [7].

42. Annulation distribution
(0) limbs only
(1) trunk and limbs
(–) inapplicable: annulations (Character 41) or paired limbs (Character 1) absent

Character 37 in Yang et al. [7].

43. Organization of trunk annulation
(0) homonomous
(1) heteronomous
(–) inapplicable: annulations (Character 41) absent

Character 38 in Yang et al. [7].

44. Metamerically arranged dorsolateral epidermal specializations
(0) absent
(1) present

Character 39 in Yang et al. [7].

45. Nature of paired epidermal specializations
(0) epidermal depressions
(1) epidermal evaginations
(–) inapplicable: epidermal specializations (Character 44) absent

Character 40 in Yang et al. [7].

46. Proportions of epidermal trunk evaginations
(0) wider than tall (e.g. nodes or plates)
(1) taller than wide (e.g. spines)
(–) inapplicable: epidermal evaginations (Character 45) absent

Character 41 in Yang et al. [7].

47. Trunk epidermal evaginations with acute distal termination
(0) absent
(1) present
(–) inapplicable: epidermal evaginations (Character 45) absent

Character 42 in Yang et al. [7].

48. Acute distal termination in epidermal evagination is curved
(0) absent
(1) present
(–) inapplicable: epidermal evaginations, if present, lack an acute distal terminus (Character 47)

Character 43 in Yang et al. [7].

49. Sclerotization of epidermal evaginations
(0) absent
(1) present
(–) inapplicable: epidermal evaginations (Character 45) absent

Character 44 in Yang et al. [7].

50. Dorsal trunk sclerite ornament
(0) net-like
(1) scaly
(–) inapplicable: sclerotized epidermal evaginations (Character 49) absent

Character 45 in Yang et al. [7].

51. Sclerites consist of a stack of constituent elements
(0) absent
(1) present
(–) inapplicable: sclerotized epidermal evaginations (Character 49) absent; terminal claws on limbs (Character 71) absent

Character 46 in Yang et al. [7].

52. Maximum number of primary dorsal epidermal specializations above each leg pair
(0) one
(1) two
(2) three
(3) four
(4) five
(5) seven
(–) inapplicable: epidermal specializations (Character 44) absent

Character 47 in Yang et al. [7].

53. Secondary sclerotized dorsolateral spines
(0) absent
(1) present
(–) inapplicable: epidermal specializations (Character 44) absent; epidermal evaginations (Character 45) absent

Character 48 in Yang et al. [7].

54. Dorsal sclerotized spine-like evaginations of variable length along the body
(0) absent
(1) present
(-) inapplicable: epidermal evaginations (Character 45) absent; epidermal evaginations taller than wide (Character 46) absent

Character 49 in Yang et al. [7].

55. Spacing between dorsolateral epidermal specializations along longitudinal body axis
(0) epidermal specializations regularly spaced
(1) epidermal specializations irregularly spaced
(–) inapplicable: epidermal specializations (Character 44) absent

Character 50 in Yang et al. [7].

56. Papillae on trunk annulations
(0) absent
(1) present
(–) inapplicable: annulations (Character 41) absent

Character 51 in Yang et al. [7].

57. Serially repeated mid-gut glands
(0) absent
(1) reniform, submillimetric lamellar

Character 52 in Yang et al. [7].

58. Fused posterior shield [NEW]
(0) absent
(1) present
(–) inapplicable: sclerotized dorsal integument with arthrodial membranes (Character 37) absent

59. Cirri
(0) absent
(1) present
(–) inapplicable: sclerotized dorsal integument with arthrodial membranes (Character 37) present

Character 95 in Yang et al. [2].

60. Trunk appendages
Trunk exites
(0) absent
(1) present
(–) inapplicable: paired appendages (Character 1) absent

Character 53 in Yang et al. [7].

61. Exite organization
(0) lanceolate dorsal blades
(1) simple oval paddle with marginal spines
(2) bipartite shaft with lamellar setae
(3) numerous podomeres, each bearing a single setae
(4) books gills
(–) inapplicable: trunk exites (Character 60) absent

Character 54 in Yang et al. [2].

62. Exites/lanceolate dorsal blades associated with dorsolateral flaps
(0) absent
(1) present
(-) inapplicable: trunk exites (Character 60) absent

Character 55 in Yang et al. [7].

63. Exite/setal blade distribution
(0) confined laterally
(1) present dorsally
(2) internalized into body cavity
(-) inapplicable: exites (Character 60) absent; dorsal integument sclerotized (Character 37) present.

Character 56 in Yang et al. [7].

64. Dorsal flaps/exites fused with endopod into biramous appendage
(0) not fused
(1) fused
(–) inapplicable: trunk exites (Character 60) absent

Character 57 in Yang et al. [7].

65. Antero-posteriorly compressed protopodite with gnathobasic endites in post-deutocerebral appendage pair
(0) absent
(1) present
(–) inapplicable: post-ocular appendages (Character 9) not arthropodized

Character 58 in Yang et al. [7].

66. Secondary structures on lobopodous limbs
(0) absent
(1) present
(–) inapplicable: post-ocular appendages (Character 9) not lobopodous

Character 59 in Yang et al. [7].

67. Nature of secondary structures
(0) spines/setae
(1) appendicules
(–) inapplicable: secondary structures on the lobopodous limbs (Character 66) absent

Character 60 in Yang et al. [7].

68. Length of spines on lobopodous limbs
(0) short (e.g. Aysheaia, Diania)
(1) long (i.e. Luolishaniidae)
(–) inapplicable: spines on lobopodous limbs (Character 67) absent or inapplicable

Character 61 in Yang et al. [7].

69. Papillae on lobopodous limbs
(0) absent
(1) present
(–) inapplicable: limbs (Character 9) are not lobopodous

Character 62 in Yang et al. [7].

70. Finger-like elements in distal tip of limbs
(0) absent
(1) present
(–) inapplicable: paired appendages (Character 1) absent

Character 63 in Yang et al. [7].

71. Terminal claws on trunk limbs
(0) absent
(1) present
(–) inapplicable: paired appendages (Character 1) absent

Character 64 in Yang et al. [7].

72. Terminal claws with multiple branches
(0) absent
(1) present
(–) inapplicable: terminal claws (Character 71) absent

Character 65 in Yang et al. [7].

73. Number of claws on trunk limbs
(0) one
(1) two
(2) three
(3) four
(4) six
(5) seven
(–) inapplicable: terminal claws (Character 71) absent

Character 66 in Yang et al. [2].

74. Differentiated distal foot in lobopodous trunk limbs
(0) absent
(1) present
(–) inapplicable: paired appendages (Character 1) absent; post-ocular appendages sclerotized (Character 9); inner branch modified as lateral flaps (Character 12).

Character 67 in Yang et al. [7].

75. Hypertrophied set of anterior body flaps
(0) absent
(1) present
(–) inapplicable: inner branch is not a lateral flap (Character 12) and dorsolateral flaps (Character 62) absent

Character 68 in Yang et al. [7].

76. Strengthening rays in lateral flaps
(0) absent
(1) present
(–) inapplicable: inner branch is not a lateral flap (Character 12) and dorsolateral flaps (Character 62) absent

Character 69 in Yang et al. [7].

77. Posterior tapering of lateral flaps
(0) absent
(1) present
(–) inapplicable: inner branch is not a lateral flap (Character 12) and dorsolateral flaps (Character 62) absent

Character 70 in Yang et al. [7].

78. Anterior sets of reduced lateral flaps
(0) absent
(1) present
(–) inapplicable: inner branch is not a lateral flap (Character 12) and dorsolateral flaps (Character 62) absent

Character 71 in Yang et al. [7].

79. Lobopodous limbs differentiated into two batches of multiple anterior/long and posterior/short limbs
(0) absent
(1) present
(–) inapplicable: post-ocular appendages (Character 8) not lobopodous

Character 72 in Yang et al. [7].

80. Number of anterior morphologically differentiated elongated limbs
(0) five (*Luolishania, Acinocricus*, Collins' monster EBS)
(1) six (*Collinsium, Collinsovermis*)
(–) inapplicable: paired appendages (Character 1) absent; morphologically distinct appendage batches (Character 79) absent

Character 73 in Yang et al. [7].

81. Appendages comprise 15 or more podomeres
(0) Fewer than 15 podomeres
(1) 15 or more podomeres
(–) inapplicable: post-ocular appendages (Character 9) not arthropodized

Character 74 in Yang et al. [7].

Posterior termination

82. Limbless posterior extension of the lobopodous trunk beyond last appendage pair
(0) absent
(1) present
(–) inapplicable: paired appendages (Character 1) absent; dorsal trunk covered by sclerotized plates (Character 35) present

Character 75 in Yang et al. [7].

83. Posterior tagma composed of three paired lateral flaps
(0) absent
(1) present
(–) inapplicable: inner branch is not a lateral flap (Character 12) and dorsolateral flaps (Character 62) absent

Character 76 in Yang et al. [7].

84. Posteriormost trunk appendage pair structurally differentiated
(0) undifferentiated
(1) differentiated
(–) inapplicable: paired appendages (Character 1) absent

Character 77 in Yang et al. [7].

85. Nature of differentiated posterior appendages
(0) appendicular tail
(1) partially fused/reduced walking legs
(–) inapplicable: posterior appendages undifferentiated (Character 84)

Character 78 in Yang et al. [7].

86. Nature of appendicular tail
(0) tail rami
(1) tail flaps
(–) inapplicable: appendicular tail (Character 85) absent

Character 79 in Yang et al. [7].

87. Claws on posterior appendages directed anteriad
(0) normal orientation (claws pointing posteriad)
(1) rotated anteriad
(–) inapplicable: appendages lack terminal claws (Character 71); appendicular tail (Character 85) present

Character 80 in Yang et al. [7].

Cardiovascular and neurological organization

88. Dorsal heart
(0) absent
(1) present

Character 81 in Yang et al. [2].

89. Dorsal condensed brain
(0) absent
(1) present

Character 82 in Yang et al. [2].

90. Number of neuromeres integrated into the dorsal condensed brain
(0) one
(1) two
(2) three
(–) inapplicable: dorsal condensed brain (Character 88) absent

Character 83 in Yang et al. [2].

91. Mouth innervation relative to brain neuromeres
(0) protocerebral innervation
(1) deutocerebral innervation
(2) tritocerebral innvervation
(3) innervation from multiple neuromeres
(–) inapplicable: dorsal condensed brain (Character 88) absent

Character 84 in Yang et al. [2].

92. General organization of VNC
(0) paired
(1) unpaired

Character 85 in Yang et al. [2].

93. VNC with morphologically discrete condensed hemiganglia connected by median commisures
(0) absent
(1) present

Character 86 in Yang et al. [2].

94. Paired nerve cord lateralized
(0) absent
(1) present
(–) inapplicable: VNC unpaired (Character 92)

Character 87 in Yang et al. [2].

95. Paired nerve cord with median interpedal commissures
(0) absent
(1) present
(–) inapplicable: VNC unpaired (Character 92)

Character 88 in Yang et al. [2].

96. Nerve cord with orthogonal organization
(0) absent
(1) present

Character 89 in Yang et al. [2].

97. Orthogonal nerve cord with ring-commissures
(0) absent
(1) present
(–) inapplicable: VNC organization not orthogonal (Character 96)

Character 90 in Yang et al. [2].

98. Segmental nerves shifted anteriorly relative to appendages, following parasegmental organization
(0) absent
(1) present
(–) inapplicable: paired appendages (Character 1) absent

Character 91 in Yang et al. [2].

99. Paired segmental leg nerves
(0) absent
(1) present
(–) inapplicable: paired appendages (Character 1) absent

Character 92 in Yang et al. [2].

100. Regularly spaced peripheral nerves running entire length of nerve cord
(0) absent
(1) present

Character 93 in Yang et al. [2].

101. Stomatogastric ganglion
(0) absent
(1) present

Character 94 in Yang et al. [2].

102. Prosomal synganglion [NEW]
(0) absent
(1) present

A synganglion, consisting of the fusion of multiple anterior segments into a single functional neurological unit, is expressed in the megacheiran *Alalcomenaeus* and extant chelicerates, including *Limulus* [1, 10].

103. Posterior longitudinal connectives [NEW]
(0) absent
(1) present

Posterior longitudinal connectives are expressed in the trunk of the megacheiran *Alalcomenaeus* and extant chelicerates, including *Limulus* [1, 10].

104. Extended oesophageal foramen [NEW]
(0) absent
(1) present

An extended oesophageal forman, namely the opening in the VNC that accommodates the downward facing J-shaped foregut in euarthropods, is present in the megacheiran *Alalcomenaeus* (Supplementary Figure S1) and extant chelicerates, including *Limulus* [1, 10].

105. Optic neuropil outside protocerebrum [NEW]
(0) absent
(1) present
(–) inapplicable: eyes (Character 30) absent

Character 143 in Tanaka et al. [1]. A single optic neuropil outside the protocerebrum, connected to the eye doublets, is known from the megacheiran *Alalcomenaeus* and *Limulus* [1].

106. Number of nested optic neuropils [NEW]
(0) two
(1) three
(2) four
(–) inapplicable: eyes (Character 30) absent

Characters 123, 124 and 125 in Tanaka et al. [1]. Three optic neuropils have been described for *Mollisonia plenovenatrix* [3] and the fuxianhuiid *Fuxianhuia protensa* [11, 12], whereas two neuropils are known from the radiodont *Lyrarapax unguispinus* [13], the megacheiran *Alalcomenaeus* (Supplementary Figure 1) and also extant euchelicerates [1].

Primary references for palaeoneuroanatomical information in Cambrian euarthropods

- *Kerygmachela kierkegaardi* [14]
- *Lyrarapax unguispinus* [13]
- *Fuxianhuia protensa* [11, 12]
- *Chengjiangocaris kunmingensis* [2]
- *Alalcomenaeus* sp. [1, 10]
- *Mollisonia plenovenatrix* [3]
- *Mollisonia symmetrica* [this study]

Supplementary Discussion

Preservation of mollisoniid neurological tissues

Both MCZ 1181 (Fig. 1, 2; Supplementary Fig. 1) and USNM 305093 (Fig. 3, Supplementary Fig. 2) contain details of the internal anatomy preserved as carbonaceous remains, sometimes associated with authigenic minerals (e.g. apatite), in accordance with the model explaining the preservation of labile tissues in the Burgess Shale [15, 16]. The expression of neuroanatomical structures as carbonaceous traces is also consistent with previous analyses of other fossils from these remarkable beds and similar Cambrian deposits in North America and South China [1, 2, 10, 11, 12, 13, 14]. Although our studied specimens show complementary dorsal and lateral views, they share several key taphonomic attributes. Bulbous lateral eyes are present in both MCZ 1181 and USNM 305093, which protrude anteriorly beyond the head shield, and feature a light-coloured outer layer (eye lens) and dark-coloured inner core (retina). These ocular characteristics are also confirmed by a third specimen of *Mollisonia symmetrica* (USNM 57661) from the Smithsonian collections (Supplementary Fig. 4). The internal organization of the lateral eyes in mollisoniids – including the putatively distinct species *Mollisonia plenovenatrix* [3] – parallels that of other non-biomineralized euarthropods from the Burgess Shale such as *Odaraia alata* [17] and *Waptia fieldensis* [18, 19]. A similar motif has also been observed in euarthropods with stalked eyes from stratigraphically older (Stage 3) Chengjiang and Xiaoshiba biotas in South China, namely radiodonts [14], fuxianhuiids [11, 12], megacheirans [1], and non-biomineralizing trilobitomorphs [20]. In both studied *Mollisonia* specimens, the lateral eyes also feature well-defined optic nerves that converge into a carbonaceous mass within the head shield (Fig. 2, 3c; Supplementary Figs. 1 and 2), indicating that this region contains legitimate neuroanatomical information.

Recent taphonomic studies have emphasized caution in the interpretation of decay-prone tissues in Cambrian ecdysozoan fossils [21, 22, 23, 24]. Several Burgess Shale euarthropods contain serially repeated axial structures, sometimes preserved as reflective carbon films, which are better interpreted as internal body cavities that have been partially filled up with microbe-rich decay fluids after rupture of the gut [19, 22, 25, 26]. We can discard this interpretation in our studied material based on a combination of anatomical and taphonomic criteria [10]. MCZ 1811 shows clear morphological and compositional differences between the preserved neurological structures (i.e. the lateral eyes, optic nerves, and cephalic CNS) and the wider (trans.) phosphatized gut tract with paired diverticulae that extends through most of the trunk (Fig. 1, 2; Supplementary Fig. 1). The relative position of these nervous and digestive structures is also in accordance with the anatomy of modern taxa. The euarthropod gut is located dorsal to the CNS through most of the body, except at the level of the mouth

where it curves ventrally, passes through the oesophageal foramen and opens on the ventral surface, whereas the CNS extends further forwards to reach the eyes [5]. The occurrence of the carbonaceous traces in the anterior third of the head only is consistent with them representing the optic nerves and the brain, the rest of the CNS (the VNC) being concealed under the gut posteriorly (Fig. 2). In USNM 305093, the interpretation of the axial reflective strand as the CNS is supported by its connections to the eyes via the optic nerves, its complex organization as a chain of metameric ganglia, and the bifurcating segmental nerves that extend ventrally beyond the limits of the dorsal exoskeleton (Fig. 3; Supplementary Fig. 2). The absence of trunk limbs in USNM 305093 and the delicate nature of the internal reflective films and their connection to the eyes are inconsistent with their interpretation as biofilm replicas of body cavities similar to those described in other BST fossils [21, 22, 25]. We also rule out the possibility that this complex axial reflective strand may be composed of both CNS and gut remains, given that the structure only extends to the eyes anteriorly (no branch towards the ventral surface), occupies a particularly ventral position, only includes simple thread-like structures extending ventrally from the ganglion-like masses, and lacks a ventral extension posteriorly. Mollisoniid digestive system (Fig. 1, 2; Supplementary Fig. 4; see also ref. [3, 8]) differs markedly from the structure exhibited by USNM 305093 in various ways; it consists of a series of at least 11 intersegmentally disposed pairs of large ovoid gut diverticulae, which insert broadly on the sides of a particularly thick gut tract that occupies a more central position compared to the VNC (compare Fig. 3 and Supplementary Fig. 4), and noticeably tapers posteriorly (Fig. 2b). Thus, the presence of CNS preservation in MCZ 1811 and USNM 305093 is supported by multiple criteria that include bilateral symmetry, morphological fidelity, carbonaceous preservation of labile tissues, position relative to other anatomical features, and congruence between specimens [10].

Supplementary References

1. Tanaka, G., Hou, X., Ma, X., Edgecombe, G.D. & Strausfeld, N.J. Chelicerate neural ground pattern in a Cambrian great appendage arthropod. Nature **502,** 364-367 (2013).
2. Yang, J., Ortega-Hernández, J., Butterfield, N.J., Liu, Y., Boyan, G.S., Hou, J.B., Lan, T. & Zhang, X.G. Fuxianhuiid ventral nerve cord and early nervous system evolution in Panarthropoda. PNAS **113,** 2988-2993 (2016).
3. Aria, C. & Caron, J.B. A middle Cambrian arthropod with chelicerae and proto-book gills. Nature **573**, 586-589 (2019).
4. Ortega-Hernández, J. 2016. Making sense of 'lower' and 'upper' stem-group Euarthropoda, with comments on the strict use of the name Arthropoda von Siebold, 1848. Biol. Rev. **91,** 255-273 (2016).
5. Ortega-Hernández, J., Janssen, R. & Budd G.E. Origin and evolution of the panarthropod head–a palaeobiological and developmental perspective. Arthrop. Struc. Dev. **46**, 354-379 (2017).
6. Smith, M.R. & Ortega-Hernández, J. *Hallucigenia's* onychophoran-like claws and the case for Tactopoda. Nature **514**, 363-366 (2014).
7. Yang, J., Ortega-Hernández, J., Gerber, S., Butterfield, N.J., Hou, J.B., Lan, T. & Zhang, X.G. A superarmored lobopodian from the Cambrian of China and early disparity in the evolution of Onychophora. PNAS **112**, 8678-8683 (2015).
8. Lerosey-Aubril, R., Skabelund, J. & Ortega-Hernández, J. Revision of the mollisoniid chelicerate (?) *Thelxiope*, with a new species from the middle Cambrian Wheeler Formation of Utah. PeerJ **8**, e8879 (2020).
9. Liu, Y., Ortega-Hernández, J., Zhai, D. and Hou, X., 2020. A reduced labrum in a Cambrian great-appendage Euarthropod. Curr. Biol. **30**, 3057-3061.
10. Ortega-Hernández, J., Lerosey-Aubril, R. & Pates, S. Proclivity of nervous system preservation in Cambrian Burgess Shale-type deposits. Proc. Roy. Soc. B, **286**, 20192370 (2019).
11. Ma, X., Hou, X., Edgecombe, G.D. and Strausfeld, N.J. Complex brain and optic lobes in an early Cambrian arthropod. Nature **490**, 258-261 (2012).
12. Ma, X., Edgecombe, G.D., Hou, X., Goral, T. & Strausfeld, N.J. Preservational pathways of corresponding brains of a Cambrian euarthropod. Curr. Biol. **25,** 2969-2975 (2015).
13. Cong, P., Ma, X., Hou, X., Edgecombe, G.D. & Strausfeld, N.J. Brain structure resolves the segmental affinity of anomalocaridid appendages. Nature **513**, 538-542 (2014).
14. Park, T.Y.S., Kihm, J.H., Woo, J., Park, C., Lee, W.Y., Smith, M.P., Harper, D.A., Young, F., Nielsen, A.T. & Vinther, J. Brain and eyes of *Kerygmachela* reveal protocerebral ancestry of the panarthropod head. Nat. Commun. **9**, 1-7 (2018).
15. Butterfield, N.J., Balthasar, U.W.E. & Wilson, L.A. 2007. Fossil diagenesis in the Burgess Shale. Palaeontology **50**: 537-543.
16. Butterfield, N.J. 2002. *Leanchoilia* guts and the interpretation of three-dimensional structures in Burgess Shale-type fossils. Paleobiology **28:** 155-171.
17. Ortega-Hernández, J. 2015. Homology of head sclerites in Burgess Shale euarthropods. Curr. Biol. **25**: 1625-1631.
18. Strausfeld, N.J. 2016. *Waptia* revisited: intimations of behaviors. Arthrop. Struc. Dev. **45**: 173-184.
19. Vannier, J., Aria, C., Taylor, R.S. & Caron, J.B. 2018. *Waptia fieldensis* Walcott, a mandibulate arthropod from the middle Cambrian Burgess Shale. Royal Society Open Science **5**: 172206.
20. Strausfeld, N.J., Ma, X., Edgecombe, G.D., Fortey, R.A., Land, M.F., Liu, Y., Cong, P. & Hou, X. 2016. Arthropod eyes: the early Cambrian fossil record and divergent evolution of visual systems. Arthrop. Struc. Dev. **45**:152-172

21. Liu, J., Steiner, M., Dunlop, J.A. & Shu, D. 2018. Microbial decay analysis challenges interpretation of putative organ systems in Cambrian fuxianhuiids. Proc. Royal Soc. B **285**: 20180051.
22. Butler, A.D., Cunningham, J.A., Budd, G.E. & Donoghue, P.C. 2015. Experimental taphonomy of *Artemia* reveals the role of endogenous microbes in mediating decay and fossilization. Proc. Royal Soc. B **282:** 20150476.
23. Murdock, D.J., Gabbott, S.E., Mayer, G. & Purnell, M.A. 2014. Decay of velvet worms (Onychophora), and bias in the fossil record of lobopodians. BMC Evol. Biol. **14**: 1-10.
24. Sansom, R.S. 2016. Preservation and phylogeny of Cambrian ecdysozoans tested by experimental decay of *Priapulus*. Sci. Rep. **6:** 1-12.
25. Budd, G.E. & Daley, A.C. 2012. The lobes and lobopods of *Opabinia regalis* from the middle Cambrian Burgess Shale. Lethaia **45:** 83-95.
26. Aria, C. & Caron, J.B. 2015. Cephalic and limb anatomy of a new isoxyid from the Burgess Shale and the role of "stem bivalved arthropods" in the disparity of the frontalmost appendage. PLoS One **10**: e0124979.
