# Supplementary material for: Neuroanatomy in a middle Cambrian mollisoniid and the ancestral nervous system organization of chelicerates
Source: Nat Commun. 2022 Jan 20;13:410. doi: 10.1038/s41467-022-28054-9 (PMC8776822; doi:10.1038/s41467-022-28054-9)
Supplement: Supplementary file 3 — Description of Additional Supplementary Files [file 41467_2022_28054_MOESM3_ESM.pdf]

Description of Additional Supplementary Files

File Name: Supplementary Data 1
Description: Morphological character dataset for phylogenetic analyses.
