# Supplementary material for: Neuroanatomy in a middle Cambrian mollisoniid and the ancestral nervous system organization of chelicerates
Source: Nat Commun. 2022 Jan 20;13:410. doi: 10.1038/s41467-022-28054-9 (PMC8776822; doi:10.1038/s41467-022-28054-9)
Supplement: Supplementary file 5 — Reporting Summary [file 41467_2022_28054_MOESM5_ESM.pdf]

nature portfolio

Corresponding author(s): Javier Ortega-Hernandez

Last updated by author(s): Jul 10, 2021

Reporting Summary

Nature Portfolio wishes to improve the reproducibility of the work that we publish. This form provides structure for consistency and transparency in reporting. For further information on Nature Portfolio policies, see our Editorial Policies and the Editorial Policy Checklist.

Statistics

For all statistical analyses, confirm that the following items are present in the figure legend, table legend, main text, or Methods section.

n/a	Confirmed	
☒	☐	The exact sample size (*n*) for each experimental group/condition, given as a discrete number and unit of measurement
☒	☐	A statement on whether measurements were taken from distinct samples or whether the same sample was measured repeatedly
☒	☐	The statistical test(s) used AND whether they are one- or two-sided<br>*Only common tests should be described solely by name; describe more complex techniques in the Methods section.*
☒	☐	A description of all covariates tested
☒	☐	A description of any assumptions or corrections, such as tests of normality and adjustment for multiple comparisons
☒	☐	A full description of the statistical parameters including central tendency (e.g. means) or other basic estimates (e.g. regression coefficient) AND variation (e.g. standard deviation) or associated estimates of uncertainty (e.g. confidence intervals)
☒	☐	For null hypothesis testing, the test statistic (e.g. *F*, *t*, *r*) with confidence intervals, effect sizes, degrees of freedom and *P* value noted<br>*Give P values as exact values whenever suitable.*
☐	☒	For Bayesian analysis, information on the choice of priors and Markov chain Monte Carlo settings
☒	☐	For hierarchical and complex designs, identification of the appropriate level for tests and full reporting of outcomes
☒	☐	Estimates of effect sizes (e.g. Cohen's *d*, Pearson's *r*), indicating how they were calculated

Our web collection on statistics for biologists contains articles on many of the points above.

Software and code

Policy information about availability of computer code

Data collection	n/a
Data analysis	TNT (parsimony-based phylogenetic software), Mr Bayes (Bayesian inference phylogenetic software), Inkscape (vector-based graphic design freeware), Photoshop CC (licensed graphic design software).

For manuscripts utilizing custom algorithms or software that are central to the research but not yet described in published literature, software must be made available to editors and reviewers. We strongly encourage code deposition in a community repository (e.g. GitHub). See the Nature Portfolio guidelines for submitting code & software for further information.

Data

Policy information about availability of data

All manuscripts must include a data availability statement. This statement should provide the following information, where applicable:

- Accession codes, unique identifiers, or web links for publicly available datasets
- A description of any restrictions on data availability
- For clinical datasets or third party data, please ensure that the statement adheres to our policy

The studied fossil specimens are deposited at the Harvard University Museum of Comparative Zoology in Cambridge (MCZ) and the Smithsonian Institution in Washington D. C. (USNM). No new fossil specimens were collected for this study. No permissions were required for performing this research. Character coding for the phylogenetic analysis is available in the Supplementary Information. The morphological dataset is available in the Supplementary Data in both text and excel formats. Source data are provided with this paper.

Field-specific reporting

Please select the one below that is the best fit for your research. If you are not sure, read the appropriate sections before making your selection.

☐ Life sciences ☐ Behavioural & social sciences ☒ Ecological, evolutionary & environmental sciences

For a reference copy of the document with all sections, see nature.com/documents/nr-reporting-summary-flat.pdf

Ecological, evolutionary & environmental sciences study design

All studies must disclose on these points even when the disclosure is negative.

Study description	Description of fossilized neurological structures in middle Cambrian euarthropods from the Burgess Shale and analyses of their preservation and evolutionary significance.
Research sample	Fossil material from the Museum of Comparative Zoology (MCZ 1811) and the Smithsonian Institution (USNM 305093, 57661, 57663)
Sampling strategy	Visit to museum collections in search of undescribed specimens, or previously unrecognized morphological features preserved in the fossils.
Data collection	Javier Ortega-Hernandez performed visual inspection of fossil specimens at the MCZ and USNM. James Weaver produced elemental mapping analyses of specimen MCZ 1811 at the Wyss Institute, Harvard University.
Timing and spatial scale	Museum collections visited during March and April 2016 by Javier Ortega-Hernandez. Further photography performed by Javier Ortega-Hernandez during Summer 2019. Elemental mapping performed by James Weaver during Summer 2019.
Data exclusions	No data were excluded from the analyses.
Reproducibility	Fossil specimens are deposited in scientific institutions (MCZ and USNM), and available for other researchers to examine them. Original morphological dataset and character coding for the phylogenetic analysis, as well as instructions for performing the analyses, as provided in full as part of the main text and the supplementary information.
Randomization	Randomization not applicable to the study of fossil morphology nor phylogenetic analysis.
Blinding	Blinding not applicable to the study of fossil morphology nor phylogenetic analysis.

Did the study involve field work? ☐ Yes ☒ No

Reporting for specific materials, systems and methods

We require information from authors about some types of materials, experimental systems and methods used in many studies. Here, indicate whether each material, system or method listed is relevant to your study. If you are not sure if a list item applies to your research, read the appropriate section before selecting a response.

Materials & experimental systems

n/a	Involved in the study	
☒	☐	Antibodies
☒	☐	Eukaryotic cell lines
☐	☒	Palaeontology and archaeology
☒	☐	Animals and other organisms
☒	☐	Human research participants
☒	☐	Clinical data
☒	☐	Dual use research of concern

Methods

n/a	Involved in the study	
☒	☐	ChIP-seq
☒	☐	Flow cytometry
☒	☐	MRI-based neuroimaging

Palaeontology and Archaeology

Specimen provenance	Fossil material belongs to historic Burgess Shale collections held at the Museum of Comparative Zoology (MCZ 1811) and the Smithsonian Institution (USNM 305093, 57661, 57663). These collections were produced during the first half of the 20th century by Charles Doolitle Walcott (then Director of the Smithsonian Institution) and Percy Raymond (then Curator of Invertebrate Paleontology at the Museum of Comparative Zoology).
Specimen deposition	Fossil material from the Museum of Comparative Zoology (MCZ 1811) and the Smithsonian Institution (USNM 305093, 57661, 57663)

Dating methods	No new dates are provided.

☐ Tick this box to confirm that the raw and calibrated dates are available in the paper or in Supplementary Information.

Ethics oversight	No ethical approval or guidance was necessary as this work deals with existing collections in established academic institutions.

Note that full information on the approval of the study protocol must also be provided in the manuscript.
